# The new COVID-19 poor and the neglected tropical diseases resurgence

**DOI:** 10.1186/s40249-020-00784-2

**Published:** 2021-01-29

**Authors:** Peter J. Hotez, Alan Fenwick, David Molyneux

**Affiliations:** 1 Department of Pediatrics, Texas Children’s Hospital Center for Vaccine Development, National School of Tropical Medicine, Baylor College of Medicine, Houston, TX USA; 2 Department of Molecular Virology and Microbiology, Texas Children’s Hospital Center for Vaccine Development, National School of Tropical Medicine, Baylor College of Medicine, Houston, TX USA; 3 Hagler Institute for Advanced Study at Texas A&M University, College Station, TX USA; 4 Department of Biology, Baylor University, Waco, TX USA; 5 James A Baker III Institute of Public Policy, Rice University, Houston, TX USA; 6 Scowcroft Institute of International Affairs, Bush School of Government and Public Service, Texas A&M University, College Station, TX USA; 7 School of Public Health, Faculty of Medicine, Imperial College London, London, UK; 8 Centre for Neglected Tropical Diseases, Liverpool School of Tropical Medicine, Liverpool, UK

More than 100 million people are facing a return to extreme poverty because of coronavirus disease 2019 (COVID-19), while new estimates suggest that three nations—India, Nigeria, and the Democratic Republic of the Congo—may suffer the greatest economic contractions. Such findings will have profound consequences in terms of our ability to control or eliminate the most widely prevalent neglected tropical diseases

## Introduction: a new global economic setback

Beginning in the 1990s with the World Bank’s World Development Report and later, the Report of the Commission on Macroeconomics and Health, was an unprecedented recognition that the poverty-disease equation flows both ways. Poverty gives rise to disease, and disease causes poverty [1]. This principle was fundamental to one of the eight Millennium Development Goals (MDGs, namely MDG 6) in 2000: “To combat AIDS, malaria, and other diseases”, ultimately helping to form the modern framework of the neglected tropical diseases (NTDs) [2].

In 2020, the COVID-19 pandemic became a stark reminder of the links between poverty and disease. New estimates from the International Monetary Fund, the World Bank, Brookings Institution, and other organizations reveal that our 20 years of steady declines in global poverty suddenly halted and reversed. During the 2000s, the Oxford University economist Professor Paul Collier popularized the term “the bottom billion”, referring to the world’s poorest people who essentially live on no money or below the World Bank poverty line. He further highlighted how the bottom billion live in “trapped economies”, producing devastating poverty spanning generations. In part due to aggressive public health measures around MDG 6, including mass drug administration programmes for NTDs, the number of people trapped in poverty began to decline steadily.

By 2019, 650 million people lived in extreme poverty—roughly 8.4% of the global population—using as a metric families in households spending less than USD 1.90 per person per day [3, 4]. Based on the trajectories established at the start of the MDGs era in 2000, the thought was that this number might decline to just over 500 million people, or 6.3% of the world’s people over the next decade [3, 4]. However due to the economic devastation from COVID-19, there was instead an abrupt and alarming increase with more than 100 million new people thrown into extreme poverty in 2020. The estimates are that now 766 million people, or almost 10% of the global population, live as Professor Collier described more than a decade ago [3, 4].

## Expanded poverty in India and Africa

**Fig. 1:**
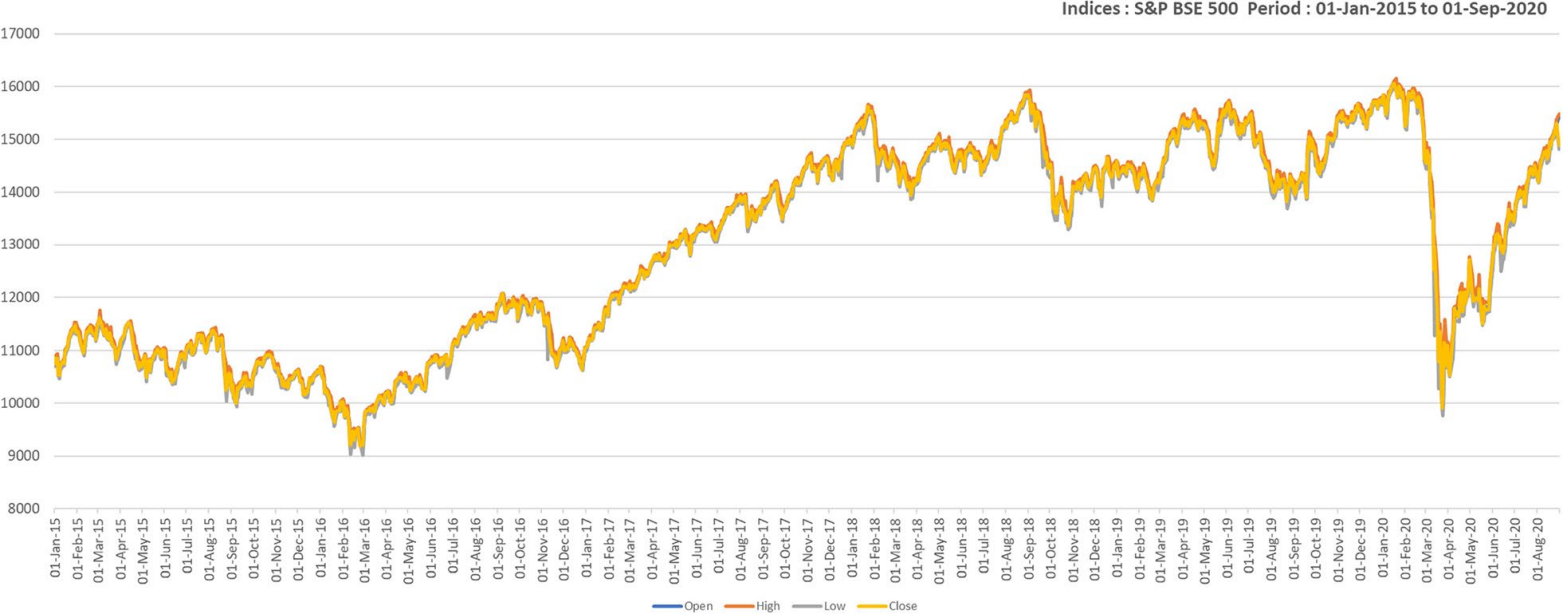
Indices: S&P Bombay Stock Exchange (BSE) 500 (1 January 2015 to 1 September 2020). https://en.wikipedia.org/wiki/COVID-19_pandemic_in_India#/media/File:Indices_S&P_BSE_500_(2015_to_2020).png

According to Homi Kharas, a senior fellow at the Center for Sustainable Development of the Brookings Institution, COVID-19 exerted its greatest economic impact in India (Fig. 1) where a segment of the population only recently escaped extreme poverty [3]. India was subsequently followed in economic reversals by Nigeria and the Democratic Republic of the Congo (DRC). Together these nations account for more than 250 million people living in extreme poverty [3]. While the population of India has suffered greatly because of COVID-19 (more than 8 million cases by November 2020) sweeping through large urban areas, neither Nigeria nor DRC experienced nearly as many COVID-19 cases. Therefore, the economic effects of COVID-19 are likely both direct and indirect. In India, the rise in poverty likely reflected aggressive social distancing mandates and closures of businesses, whereas a nation such as Nigeria is heavily dependent on the international oil and gas industry, a sector which has endured great hardships and work layoffs as a consequence of COVID-19.

Still another finding is the long-lasting effects from COVID-19 on national poverty predicted to linger after the pandemic subsides. Most of these long-term consequences are projected to again localize to Nigeria and DRC, two nations expected to eventually account for up to 40% of extreme global poverty in the coming decades according to the Bill & Melinda Gate Foundation.

According to Kharas, other countries believed to suffer long-term COVID-19 financial consequences, include Angola, Burkina Faso, Kenya, Mali, South Sudan, Tanzania, while Venezuela and Yemen represent to the two non-African countries expected to experience long term economic declines [3].

## The NTD consequences

What might we expect from the sharp rises in COVID-19-associated poverty in India, Nigeria, and DRC? In a previous study published in 2017, we found that India led the world in the total of cases of soil-transmitted helminth infections and lymphatic filariasis, as well as dengue, leprosy, rabies, cysticercosis, trachoma, and visceral leishmianiasis [5]. Similarly, Nigeria and DRC ranked first or second in schistosomiasis and onchocerciasis, as well as hosting the highest number of cases of lymphatic filariasis on the African continent [5]. Therefore, the resurgence of poverty in these countries could become a major factor in slowing global efforts to control or eliminate these conditions through preventive mass drug administration and other approaches. Moreover, the community health workers needed to administer preventive chemotherapy or other public health measures could be blocked or hampered in conducting their routine activities due to social distancing mandates, or they may be pulled into COVID-19 prevention initiatives. There is also the added complexity of agricultural declines and food insecurity from COVID-19. On the other hand, enhanced hygiene measures to prevent COVID-19, such as hand washing, might reduce the transmission of other infections, including some of the NTDs. Each of these possibilities require investigation and confirmation.

The fact that COVID-19 now disproportionately affects the economies of the world’s most endemic NTDs nations is an ominous sign. In this way, COVID-19 could derail otherwise promising control and elimination efforts for schistosomiasis, river blindness, and lymphatic filariasis in Africa or soil-transmitted helminth infections and lymphatic filariasis in India. The potential for COVID-19 to derail hard-fought NTD control and initiatives must be considered a priority for the global policy makers [6]. Focused attention on circumventing the derailment of the NTDs control and elimination ecosystem due to COVID-19 represents a new urgency. This topic must be front and centre for the World Health Assembly in 2021 or future group of 20 (G20 summits).

## Data Availability

All data is contained in the manuscript.
